# Association Between Fruit and Vegetable Intake and Skin Carotenoid Levels Among Japanese Adults in the Workplace

**DOI:** 10.3390/nu18030550

**Published:** 2026-02-06

**Authors:** Emiko Okada, Hidemi Takimoto

**Affiliations:** 1 The Health Care Science Institute, 3-2-12 Akasaka, Minato-ku 107-0052, Tokyo, Japan; 2 Department of Nutritional Epidemiology and Shokuiku, National Institute of Biomedical Innovation, Health and Nutrition, Kento Innovation Park, NK Building, 3-17 Senrioka Shinmachi, Settsu-shi 566-0002, Osaka, Japan; thidemi@nibn.go.jp

**Keywords:** skin carotenoid, fruits and vegetable intake, workplace, blood glucose, blood pressure, Japan

## Abstract

Background/Objectives: Skin carotenoid measurements have been proposed as an indicator to reflect of fruit and vegetable intake, but evidence from occupational settings remains limited. The primary aim of this study was to assess the association between fruit and vegetable intake and skin carotenoid levels in the workplace. The secondary aim was to examine the association of skin carotenoid levels with blood glucose levels and blood pressure (BP). Methods: This cross-sectional study included Japanese workers aged ≥20 years between 2022 and 2023. Skin carotenoid levels were measured, dietary intake was assessed using self-administered questionnaires, and data from workplace health check-up records were collected. Multiple regression analysis was conducted to examine the association between skin carotenoid levels and fruit and vegetable intake in 210 participants. Associations between skin carotenoid levels and log-transformed glycated haemoglobin (HbA1c), fasting blood glucose (FBG), systolic BP, and diastolic BP levels were examined in 162, 158, and 183 participants, respectively. Results: Skin carotenoid levels were positively associated with the number of vegetable dishes consumed and the frequency of fruit intake. A slight positive association was observed with HbA1c levels (partial regression coefficient = 0.00012), whereas no associations were found with FBG or BP. Conclusions: Skin carotenoid levels reflect self-reported fruit and vegetable intake, supporting their potential use as a non-invasive dietary assessment tool in workplace nutrition education. However, the associations observed with HbA1c were very small and of limited clinical significance, and the results should be interpreted with caution.

## 1. Introduction

Globally, an estimated 1.6 million deaths were directly attributed to diabetes in 2021 [1], and an estimated 1.4 billion adults had hypertension in 2024 [2]. In Japan alone, approximately 3.3 million patients with diabetes and 9.9 million with hypertensive diseases were reported in the 2017 Patient Survey [3]. Given the high burden of noncommunicable diseases associated with hyperglycaemia and elevated blood pressure (BP) [4], effective prevention strategies are urgently needed. A healthy diet plays a crucial role in preventing hyperglycaemia, raised BP, and the development of diabetes and hypertension. Epidemiological evidence suggests that a diet rich in fruits and vegetables reduces the risk of developing diabetes [5] and hypertension [6]. However, accurately assessing dietary intake can be challenging and often relies on self-reported measures that may be subject to bias.

Recently, methods using resonance Raman spectroscopy and reflection spectroscopy to detect carotenoids in human skin have gained attention as useful parameters for assessing fruit and vegetable intake [7]. Skin carotenoid levels have been shown to positively correlate with plasma or serum carotenoid concentrations [7,8,9] and to be associated with fruit and vegetable intake estimated from self-reported food frequency questionnaire surveys [10,11] or 24 h dietary recalls [12]. Skin carotenoids reflect carotenoid and fruit and vegetable intake over approximately the past 30 days [7,13]. Measurement of skin carotenoid levels is a non-invasive and objective method that minimises the burden on both participants and investigators [7]. In addition, participants can immediately see their own skin carotenoid scores, objectively “visualizing” their fruit and vegetable intake.

Given the challenges in accurately assessing dietary intake, particularly in occupational settings, non-invasive, objective, and easy to implement tools are needed. Workplaces are unique settings for nutrition assessment and education because workers spend much of their daily lives there, and the food environment influences dietary behaviours, dietary intake, and related health outcomes [14,15]. In Japan, workplace health promotion programmes, including the Specific Health Checkups and Specific Health Guidance, are implemented mainly through business establishments [16]. The working population includes both individuals who are generally healthy and those with existing health conditions; however, most workers maintain a level of health that allows continued employment. Therefore, this population represents an important target group for early prevention of noncommunicable diseases through dietary improvements, particularly increased fruit and vegetable intake. However, few studies have assessed whether the established association between fruit and vegetable intake and skin carotenoid levels holds in occupational populations, particularly regarding their applicability as a simple and non-invasive indicator for dietary assessment. Therefore, this study provides evidence on the applicability of skin carotenoid measurements among Japanese workers under practical conditions relevant to public health practice.

Previous studies examining serum carotenoid concentrations and metabolic outcomes have suggested that carotenoids may influence BP regulation and glycaemic control through their antioxidant activities and play protective roles against hypertension and impaired glucose metabolism [17,18]. Based on this biological plausibility, skin carotenoid levels as an objective indicator of fruit and vegetable intake, were hypothesised to be associated with metabolic parameters, specifically blood glucose levels and BP. Nevertheless, existing evidence remains inconsistent. A cross-sectional study of a resident-based on health check-up from 811 Japanese adults found no significant association between skin carotenoid levels and FBG levels, but suggested a negative correlation with BP in women [19]. In contrast, another cross-sectional study based on health check-up from 1812 Japanese adults reported a negative correlation between skin carotenoid levels and fasting blood glucose (FBG), but no association with glycated haemoglobin A1c (HbA1c) or BP [20]. Blood glucose and BP are routinely assessed metabolic parameters related to major modifiable risk factors. Thus, examining their associations with skin carotenoid levels in diverse populations, including occupational settings, may help to interpret findings related to skin carotenoid measurements.

This study primarily aimed to assess the association between fruit and vegetable intake and skin carotenoid levels among Japanese workers. As a secondary and exploratory aim, we examined the associations between skin carotenoid levels and metabolic parameters, specifically blood glucose levels and BP.

## 2. Materials and Methods

### 2.1. Study Population

This cross-sectional study was conducted among workers at 12 business establishments in Gero, Gifu, Japan, from November 2022 to February 2023. The study included all 12 workplaces certified as Gero City Health Promotion Businesses by the local government in 2022, comprising five manufacturing companies (e.g., metal processing, automobile parts, furniture, automobile maintenance), three construction companies, two branches of financial institutions, one transportation company, and one electrical construction company. Participants were recruited from employees at each workplace using a convenience sampling method. The inclusion criteria were employees aged 20 years or older who were at work on the day of the survey, and there were no specific exclusion criteria. Participants were recruited from several months to a few weeks before the survey through workplace staff at each business establishment. The study was announced via posters displayed within and circulated throughout the workplace, as well as emails outlining the study overview. On the day of the survey, researchers invited employees to participate at a designated location within each establishment (e.g., a meeting room). After providing both oral and written explanations of the study by researchers, those who agreed to participate were included. Participation in the study was voluntary, and informed consent was obtained from all participants using a tablet device. Because participants were recruited voluntarily from workplaces certified as Health Promotion Businesses by the local government, the study population may include healthier workers and may systematically differ from the general working population, and the possibility of selection bias should be considered. Of the 1157 employees from the 12 sites, a total of 210 who met the inclusion criteria participated in the study. The skin carotenoid levels of the participants were measured, and they completed a self-administered questionnaire survey. Additionally, 185 participants provided information on the results of the most recent health check-ups conducted at their workplaces. Information on HbA1c, FBG, and BP levels was obtained from 162, 158, and 183 participants, respectively. This study was conducted in accordance with the Declaration of Helsinki and the Ethical Guidelines for Medical and Health Research Involving Human Subjects. The study protocol was approved by the Ethics Review Committees of the National Institute of Biomedical Innovation, Health, and Nutrition (approval number: KENEI 197 and date of approval: 26 September 2022) and the Health Care Science Institute (approval number: 2023-1 and date of approval: 24 July 2023).

### 2.2. Measurement of Skin Carotenoid Levels

Skin carotenoid levels were measured using the Veggie Meter^®^ (Longevity Link Japan Corporation, Yokohama, Kanagawa, Japan). Measurement of skin carotenoid levels using reflectance spectroscopy with the Veggie Meter^®^ has demonstrated validity, as indicated by a high linear correlation with total serum carotenoid concentrations (R = 0.81) [7]. Measurement reproducibility showed a relative standard deviation of 3.4–4.1%, indicating precision [7]. This measurement method has been validated for use in adults to determine carotenoid levels in the skin [7]. The operational principles of this device have been described previously [13]. Briefly, the Veggie Meter^®^ objectively assesses carotenoid pigment concentrations in the human skin via pressure-mediated reflection spectroscopy. The device was calibrated daily using the supplied dark and white reference standards prior to the measurements. Participants disinfected their fingers using disposable wipes before measurement. The middle finger of the left hand was then placed in the finger cradle and pressed against the convex contact lens with the spring-loaded cover. Measurements were obtained using the device’s averaging mode, and the mean of three consecutive readings was recorded as the skin carotenoid score. Skin carotenoid scores are unitless and range from 0 to 1200.

### 2.3. Dietary Survey

A self-administered questionnaire using a tablet device was used to assess the number of vegetable dishes consumed, fruit intake, and the frequency of food intake. One vegetable serving was defined as 70 g based on previous studies [21,22]. The number of vegetable dishes (i.e., servings) was evaluated across six categories: almost never, 1, 2, 3, 4, and more than or equal to 5 dishes per day. Food intake frequency over the past month was assessed using a modified version of the 2013 National Health and Nutrition Survey questionnaire in Japan [23], with additional items on vegetable and fruit juice and milk and dairy product intake. Based on this classification, the 14 food items included rice, bread, noodles, meat, fish and shellfish, eggs, soybeans and soybean products, green and yellow vegetables, light-coloured vegetables, vegetable and tomato juice, green juice with kale, fruits, 100% fruit juice, and milk and dairy products. Green and yellow vegetables mainly include dark-coloured vegetables such as tomatoes, carrots, spinach, and bell peppers, whereas light- coloured vegetables mainly include vegetables such as cabbage, cucumbers, radishes, and onions, based on the National Health and Nutrition Survey questionnaire in Japan. These food items were assessed using five frequency categories: more than or equal to 1 time/day, 4 to 6 times/week, 2 to 3 times/week, 1 time/week, and less than 1 time/week. Additionally, information on the use of specific dietary supplement related to carotenoids was collected, and responses were categorised as never, rarely, sometimes, or every day. Dietary supplement use included lutein/zeaxanthin, β-carotene/carotenoids, lycopene, astaxanthin, coenzyme Q10, multivitamins/vitamins A, C, and E, spirulina, chlorella, blueberries, vegetable supplements, and others.

### 2.4. Survey on Demographics and Behavioural Factors

Information on demographic and behavioural factors was collected through a self-administered questionnaire using a tablet device after completion of the dietary survey. The collected information included sex, age, height, weight, smoking status (never-smoker, former smoker, occasional smoker, or smoker), passive smoking status (never, once a month, once a week, several times a week, or almost every day), alcohol consumption status (non-drinker, drinking 1–3 times/month, 1–2 times/week, 3–4 times/week, 5–6 times/week, or everyday), physical activity level, sleeping hours (<5, 5–<6, 6–<7, 7–<8, 8–<9, or ≥9 h/day), medical history and current illnesses (diabetes, hypertension, hyperlipidaemia, cancer, cardiovascular disease, respiratory disease, gastrointestinal, liver, and gall bladder diseases, none, or other). Physical activity level was assessed based on the 2017 National Health and Nutrition Survey questionnaire in Japan [24], including sitting time (<3, 3–8, or ≥8 h/day), time spent walking or standing (<1, 1–3, or ≥3 h/day), and time spent doing physical labour, such as carrying loads (none, <1, or ≥1 h/day). Body mass index (BMI) was calculated using self-reported weight (kg) divided by height in metres squared (m) (kg/m^2^).

### 2.5. Assessment of Blood Glucose Levels and BP

Information on HbA1c levels, FBG levels, systolic blood pressure (SBP), and diastolic blood pressure (DBP) was obtained from the results of the most recent health check-ups conducted at each participants’ workplace, one to nine months prior to the measurement of skin carotenoid levels. Health check-ups in Japan are conducted under the Industrial Safety and Health Law using standardised protocols, including blood sampling after an overnight fast of at least 10 h and BP measurement after a period of rest in the seated position, with common test items and methods applied nationwide [25]. The mean values of the SBP and DBP measurements were used.

### 2.6. Statistical Analysis

Outliers were identified using the interquartile range; however, they were retained in the analysis because all values flagged as outliers were reviewed and found to be clinically plausible and within ranges reported in previous study [26]. In relatively small samples with a narrow distribution, such values may reflect real characteristics within the study population rather than measurement or recording errors. First, to explore the association between skin carotenoid levels and the characteristics and dietary intake of the participants, the Mann–Whitney U test or Kruskal–Wallis test was used for comparisons between two or three groups for mean skin carotenoid levels. Spearman’s rank correlation coefficient was used to assess associations between continuous variables. Second, linear regression analysis was used to examine the association between fruit and vegetable intake (categorical variables) as the explanatory variable and skin carotenoid levels as the outcome variable, using both crude and multivariate models. To confirm the trend, models were also conducted treating fruit and vegetable intake as continuous variables. Variables for vegetable and fruit intake included number of vegetable dishes consumed (almost never, 1, 2, 3, or ≥4 dishes/day) and frequency of fruit or green and yellow vegetables or light-coloured vegetables intake (<1 time/week, 1 time/week, 2–3 times/week, 4–6 times/week, or ≥1 time/day). Each variable was entered into the models separately, with the lowest category used as the reference. The multivariate models were adjusted for sex, age group (20–39, 40–49, 50–59, or ≥60 years), BMI (<18.5, 18.5–24.9, or ≥25.0 kg/m^2^), smoking status (non-smoker or smoker), alcohol drinking status (non-drinker or drinker), and use of any dietary supplement (yes or no). Since the number of participants taking each specific supplement was small, dietary supplement variables were merged and analysed as a single binary variable indicating any dietary supplement use, to avoid model instability due to sparse data. Covariates for the adjusted model were selected based on previous studies [27] and the factors affecting the outcome and explanatory variables. Finally, linear regression analysis was conducted to assess the association between skin carotenoid levels as the explanatory variable and blood glucose and BP levels as the outcome variable, using both crude and multivariate models. Blood glucose and BP measurements were not normally distributed; therefore, a logarithmic transformation was used in the analysis. Multivariate models were adjusted for sex, age group, BMI, smoking status, alcohol consumption status, dietary supplement use, and history of diabetes or hypertension (yes or no). To assess potential nonlinear relationships between skin carotenoid scores and health-related variables, restricted cubic spline analysis with three knots was conducted. As no evidence of nonlinearity was observed, linear regression analysis was subsequently applied in this study. All statistical analyses were performed using the SAS statistical package for Windows version 9.4 (SAS Institute Inc., Cary, NC, USA). Statistical significance was set at a *p*-value of less than 0.05.

## 3. Results

The median (25th–75th percentile) skin carotenoid level of all participants was 322 (262–391). The mean and standard deviation (SD) of skin carotenoid levels according to participant characteristics are shown in Table 1. Skin carotenoid levels were higher among women, older participants, those with lower BMI, dietary supplement consumers, and non-smokers.

The mean (SD) skin carotenoid levels according to dietary intake status are presented in Table 2. Skin carotenoid levels were higher among participants with less frequent consumption of noodles and meat and among those with more frequent consumption of fish and shellfish, vegetables, fruits, and tomato juice.

The results of the linear regression analysis of the association between fruit and vegetable intake and skin carotenoid levels are shown in Table 3. The number of vegetable dishes and the frequency of fruit intake were positively associated with skin carotenoid levels, showing a dose–response relationship. In contrast, the frequency of green and yellow vegetables and light-coloured vegetables was not significantly associated with skin carotenoid levels.

The median (25th–75th percentile) values for HbA1c, FBG, SBP, and DBP were 5.40 (5.20–5.70) %, 92.0 (85.0–100) mg/dL, 121 (112–135) mmHg, and 77.0 (69.0–84.7) mmHg, respectively. Table 4 shows the results of the linear regression analysis examining the association between skin carotenoid levels and log-transformed HbA1c levels, FBG levels, SBP, and DBP levels. Skin carotenoid levels showed a slight positive association with HbA1c levels (partial regression coefficient in the adjusted model: 0.00012). This corresponds to an approximately 0.012% relative increase in HbA1c per one-unit increase in skin carotenoid levels, equivalent to a 0.065% increase per 100-unit increase. However, FBG, SBP, and DBP levels were not significantly associated with skin carotenoid levels.

## 4. Discussion

This study examined the association between fruit and vegetable intake and skin carotenoid levels among Japanese workers and explored associations between skin carotenoid levels and metabolic parameters. We found that the self-reported number of vegetable dishes consumed and the frequency of fruit intake were positively associated with skin carotenoid levels. However, skin carotenoid levels showed a slight positive association with HbA1c levels, whereas no associations were observed with FBG or BP.

In this study, overall fruit and vegetable intake was associated with higher skin carotenoid levels, whereas no associations were observed for vegetable colour categories. Numerous studies have shown an association between higher self-reported fruit and vegetable intake and higher skin carotenoid levels [10,11,28,29,30]. Previous research has also shown that skin carotenoid scores correlate strongly with plasma concentrations of major dietary carotenoids commonly found in green and yellow vegetables, including α-carotene, β-carotene, β-cryptoxanthin, lutein, and zeaxanthin, but not lycopene [31]. Absorption and deposition of carotenoids differ by species, and these differences may not be fully reflected in skin carotenoid measurements. In addition, while the number of vegetable dishes allowed an approximate estimation of intake amount (one dish defined as 70 g) in this study, vegetable intake by colour category was assessed only by frequency, which may have influenced the observed associations with skin carotenoid levels. Furthermore, dietary intake was assessed using a self-reported questionnaire based on food frequency, and it is important to note that the possibility of information bias due to recall bias cannot be ruled out. Taken together, these factors suggest that the observed associations likely reflect the cumulative intake of various carotenoids from a diverse range of fruits and vegetables rather than the contribution of specific vegetable types or individual carotenoid species. In Japan, five vegetable dishes corresponded to the recommended daily vegetable intake of 350 g for adults under the Health Japan 21 (second term) [32]. However, only a small proportion of participants in this study met higher intakes of vegetable dishes or fruit, suggesting that overall fruit and vegetable consumption may have been inadequate in this working population. Given this context, the ease of use of skin carotenoid measurement devices may be particularly useful in workplace settings to raise awareness of fruit and vegetable intake and support dietary improvement among employees.

In this study, a slight positive association was observed between skin carotenoid levels and HbA1c, whereas no associations were found with FBG. HbA1c reflects average glycaemic exposure over the preceding one to two months, and skin carotenoid levels also represent dietary intake over a similar time frame [7,13]. This temporal characteristic may partly explain why an association was observed with HbA1c but not with FBG, which reflects short-term glycaemic status. The direction of this association was unexpected. Previous serum-based studies have shown inverse associations between carotenoid concentrations and HbA1c levels [33], FBG, type 2 diabetes, impaired glucose metabolism [34], and homeostasis model assessment-insulin resistance [35]. One possible explanation is residual confounding or reverse causality, whereby individuals with higher HbA1c levels may have increased their fruit and vegetable intake, leading to higher skin carotenoid levels. In addition, a meta-analysis of prospective studies reported a nonlinear association between fruit and vegetable intake and the risk of type 2 diabetes [36]. Concerns have also been raised about the high sugar content of fruits, particularly fructose [37]; however, an intervention trial showed no significant changes in HbA1c or blood glucose levels after fruit supplementation [38]. The association between carotenoids measured in the skin and blood glucose levels has not been consistent in the literature [19,20]. Differences in the measurement devices may also contribute to discrepant findings. Several spectroscopy-based devices with distinct characteristics are used to measure skin carotenoid levels, with some directly capturing carotenoids across different spectral regions and others estimating skin pigment colour as a proxy [39]. Seasonal variation may be another factor. Skin carotenoid levels can fluctuate with season due to changes in fruit and vegetable consumption and sun exposure, with some studies reporting lower levels in winter compared to summer and fall [27]. In the present study, skin carotenoid measurements were conducted from November to February, during the winter season in Japan, which may have influenced the observed association with HbA1c. Considering the very small effect size observed and the fact that the range of HbA1c values was largely within the normal clinical range, this finding is unlikely to have meaningful implications for glycaemic control in a generally healthy working population. Therefore, although the association reached statistical significance, its clinical relevance may be limited, and careful interpretation of the results is warranted.

Serum carotenoid levels have been associated with a low prevalence of hypertension [40] and a low risk of mortality from cardiovascular disease [41,42]. Furthermore, a meta-analysis of randomised controlled trials has reported beneficial effects of carotenoid supplementation on BP parameters [17]. By contrast, in the present study, carotenoids measured in the skin were not associated with BP, and this null finding should be interpreted with caution. This study may have been underpowered to detect associations, particularly for BP, due to the limited sample size. Our findings are consistent with a previous study that also reported no significant association between skin carotenoid levels and BP [20]. Therefore, the absence of a significant association in this study does not necessarily indicate a true lack of effect. Further longitudinal epidemiological studies with larger samples are needed to clarify the association between skin carotenoid levels and metabolic parameters, specifically blood glucose levels and BP.

From a public health perspective, this study supports the use of skin carotenoids as an objective and practical tool for assessing fruit and vegetable intake in workplace health education. Incorporating skin carotenoid measurements into workplace health programmes could enhance nutrition education and encourage healthier dietary habits. The present study, however, had a few limitations. First, owing to its cross-sectional nature, this study was unable to reveal causal associations between skin carotenoid levels, blood glucose levels, and BP. In particular, blood glucose and BP were evaluated based on the results of health check-ups conducted several months before the skin carotenoid measurements, which may not fully reflect the participants’ current health status at the time of skin carotenoid assessment. Furthermore, although standardised protocols were applied in the health check-ups, they were conducted in different workplace settings, which may have introduced potential variability in measurement conditions. In addition, participants with hyperglycaemia or elevated BP may have modified their dietary habits, such as increasing fruit and vegetable intake, which could in turn influence their skin carotenoid levels. To account for this potential confounding, diabetes or hypertension was included as a covariate in the multivariate regression models, allowing us to partially control for the effects on the observed associations. Second, this study may have been underpowered to detect associations, due to the limited sample size. In addition, undersampling further reduced the effective sample size, which may have limited the ability to detect small effects. Third, the study population consisted of individuals employed in business establishments in a single region of Japan. Although the mean of blood glucose levels and BP observed in this study were comparable to those of the general Japanese population [43], the prevalence of diabetes and hypertension in this study may have been lower than in the general population because employed individuals generally exhibit better health than unemployed individuals. Consequently, the associations between skin carotenoid levels and blood glucose levels and BP may have been underestimated, and the true magnitude of these associations in a more representative population, including unemployed individuals or those with chronic diseases, could differ from what was observed in this study. Additionally, selection bias may have occurred because the study population comprised a large proportion of men (78.6%) and individuals younger than 60 years (84.8%) (Table 1). Because this study was conducted in a single occupational group and did not include other populations or repeated measurements over time, the generalizability of the findings and their interpretation should be approached with caution. Fourth, medication use for diabetes or hypertension may influence blood glucose levels and BP. However, information on medication use was not consistently available in the present study, which may have resulted in residual confounding. Nonetheless, because we adjusted for participants’ medical history in the multivariate model, these factors are likely to have been at least partially accounted for. Fifth, dietary assessment was conducted using a self-reported questionnaire based on food frequency. Multiple-day dietary records or 24 h recalls generally provide a more precise estimate of habitual intake by reducing within-person variation; however, such detailed dietary assessments were not conducted in this study. As a result, total energy intake and nutrient composition could not be quantified. Therefore, we were unable to adjust for other dietary factors that may confound the observed associations, including total energy intake, fat intake, or major nutrient composition. Given that carotenoids are fat-soluble and their absorption may be influenced by dietary fat, residual confounding by unmeasured dietary factors cannot be ruled out. In addition, fruit intake was assessed as overall frequency, without classification by colour or carotenoid content; therefore, potential differences in associations by fruit colour could not be examined in this study. Sixth, in addition to the confounding factors considered in this study, recent sun exposure may also influence skin carotenoid levels [27]. However, sun exposure was not assessed in this study, and its potential impact requires further investigation.

## 5. Conclusions

This study indicated that among Japanese workers, skin carotenoid levels were positively associated with both the self-reported frequency of fruit intake and the number of vegetable servings consumed, supporting their potential as a practical tool for dietary assessment in workplace nutrition education. Regarding metabolic parameters, skin carotenoid levels showed a slight positive association with HbA1c levels, whereas no associations were observed with FBG or BP. However, considering the very small effect sizes and the fact that HbA1c values were largely within the normal clinical range, the clinical relevance of this association may be limited. Moreover, the statistical power may have been insufficient due to the small sample size; therefore, the results should be interpreted with caution.

## Figures and Tables

**Table 1:** Skin carotenoid levels according to participant characteristics (*n* = 210).

				Skin Carotenoid Levels		
		*n*	(%)	Mean	SD	*p*-Value
Sex	Men	165	(78.6)	333	(106)	0.039
	Women	45	(21.4)	363	(106)	
Age (years)	20–39	49	(23.3)	326	(79)	0.101
	40–49	66	(31.4)	314	(86)	
	50–59	63	(30.0)	362	(132)	
	≥60	32	(15.2)	367	(117)	
	Continuous variable			*r* = 0.140		0.043
BMI (kg/m^2^)	<18.5	11	(5.2)	354	(133)	0.182
	18.5–24.9	148	(70.5)	345	(110)	
	≥25	51	(24.3)	318	(91)	
	Continuous variable			*r* = −0.149		0.031
Dietary supplement use	Yes	56	(26.7)	410	(133)	<0.001
	No	154	(73.3)	313	(82)	
Smoking status	Smoker	55	(26.2)	310	(82)	0.037
	Non-smoker	155	(73.8)	349	(113)	
Alcohol drinking status	Drinker	137	(65.2)	333	(103)	0.271
	Non-drinker	73	(34.8)	351	(113)	
Muscular work or strenuous sports (h/day)	None	92	(43.8)	332	(88)	0.895
	<1	62	(29.5)	344	(118)	
	≥1	56	(26.7)	346	(122)	
Sedentary (h/day)	>3	48	(22.9)	352	(131)	0.659
	3–<8	139	(66.2)	338	(103)	
	≥8	23	(11.0)	318	(73)	
Walking or standing (h/day)	<1	59	(28.1)	326	(86)	0.655
	1–<3	84	(40.0)	343	(111)	
	≥3	67	(31.9)	346	(118)	
Sleeping (h/day)	<5	11	(5.2)	341	(105)	0.901
	5–<6	93	(44.3)	345	(119)	
	6–<7	83	(39.5)	330	(98)	
	7–<8	18	(8.6)	346	(97)	
	≥8	5	(2.4)	352	(66)	

BMI, body mass index; SD, standard deviation. The Mann–Whitney U test was used for comparison of means between two groups, and the Kruskal–Wallis test was used for three or more groups. Spearman’s rank correlation coefficient was used to assess associations between continuous variables.

**Table 2:** Skin carotenoid levels according to food intake status (*n* = 210).

				Skin Carotenoid Levels		
		*n*	(%)	Mean	SD	*p*-Value
Number of vegetable dishes (dish/day)	Almost never	18	(8.6)	326	(128)	0.131
	1	121	(57.6)	332	(103)	
	2	50	(23.8)	339	(84)	
	3	16	(7.6)	385	(163)	
	≥4	5	(2.4)	409	(67)	
Frequency of food group intake in the last month						
Rice	≤1 times/week	1	(0.5)	287	-	0.745
	1 times/week	3	(1.4)	312	(122)	
	2–3 times/week	10	(4.8)	324	(81)	
	4–6 times/week	18	(8.6)	314	(82)	
	≥1 time/day	178	(84.8)	343	(110)	
Breads	≤1 times/week	28	(13.3)	325	(113)	0.750
	1 times/week	64	(30.5)	332	(109)	
	2–3 times/week	41	(19.5)	338	(85)	
	4–6 times/week	21	(10.0)	351	(109)	
	≥1 time/day	56	(26.7)	351	(117)	
Noodles	≤1 times/week	21	(10.0)	347	(93)	0.011
	1 times/week	103	(49.1)	353	(125)	
	2–3 times/week	68	(32.4)	332	(81)	
	4–6 times/week	13	(6.2)	297	(77)	
	≥1 time/day	5	(2.4)	224	(21)	
Potatoes	≤1 times/week	34	(16.2)	319	(110)	0.366
	1 times/week	71	(33.8)	353	(129)	
	2–3 times/week	85	(40.5)	332	(90)	
	4–6 times/week	16	(7.6)	356	(75)	
	≥1 time/day	4	(1.9)	360	(48)	
Soybeans and soybean products	≤1 times/week	14	(6.7)	355	(128)	0.340
	1 times/week	39	(18.6)	329	(134)	
	2–3 times/week	75	(35.7)	330	(95)	
	4–6 times/week	49	(23.3)	347	(96)	
	≥1 time/day	33	(15.7)	354	(105)	
Meats	≤1 times/week	4	(1.9)	475	(72)	0.026
	1 times/week	16	(7.6)	358	(149)	
	2–3 times/week	88	(41.9)	339	(113)	
	4–6 times/week	76	(36.2)	338	(86)	
	≥1 time/day	26	(12.4)	309	(102)	
Fish and shellfish	≤1 times/week	9	(4.3)	378	(118)	0.006
	1 times/week	55	(26.2)	333	(95)	
	2–3 times/week	118	(56.2)	340	(111)	
	4–6 times/week	18	(8.6)	383	(105)	
	≥1 time/day	10	(4.8)	252	(52)	
Eggs	≤1 times/week	8	(3.8)	343	(99)	0.691
	1 times/week	23	(11.0)	317	(99)	
	2–3 times/week	88	(41.9)	340	(118)	
	4–6 times/week	43	(20.5)	347	(97)	
	≥1 time/day	48	(22.9)	340	(101)	
Green and yellow vegetables	≤1 times/week	8	(3.8)	354	(116)	0.944
	1 times/week	27	(12.9)	333	(116)	
	2–3 times/week	87	(41.4)	344	(115)	
	4–6 times/week	56	(26.7)	331	(89)	
	≥1 time/day	32	(15.2)	341	(109)	
Light coloured vegetables	≤1 times/week	12	(5.7)	277	(82)	0.031
	1 times/week	41	(19.5)	339	(111)	
	2–3 times/week	86	(41.0)	335	(89)	
	4–6 times/week	43	(20.5)	376	(130)	
	≥1 time/day	28	(13.3)	321	(109)	
Vegetable and tomato juice	≤1 times/week	152	(72.4)	329	(101)	0.013
	1 times/week	25	(11.9)	324	(91)	
	2–3 times/week	14	(6.7)	383	(146)	
	4–6 times/week	10	(4.8)	411	(118)	
	≥1 time/day	9	(4.3)	404	(108)	
Aojiru with kale	≤1 times/week	200	(95.2)	340	(108)	0.585
	2–3 times/week	2	(1.0)	260	(41)	
	4–6 times/week	5	(2.4)	331	(57)	
	≥1 time/day	3	(1.4)	338	(103)	
Fruits	≤1 times/week	39	(18.6)	291	(71)	<0.001
	1 times/week	59	(28.1)	319	(94)	
	2–3 times/week	49	(23.3)	329	(72)	
	4–6 times/week	27	(12.9)	423	(163)	
	≥1 time/day	36	(17.1)	376	(106)	
100% fruit juice	≤1 times/week	180	(85.7)	339	(109)	0.297
	1 times/week	21	(10.0)	330	(91)	
	2–3 times/week	3	(1.4)	418	(134)	
	4–6 times/week	4	(1.9)	394	(51)	
	≥1 time/day	2	(1.0)	271	(95)	
Milk and dairy products	≤1 times/week	46	(21.9)	313	(87)	0.205
	1 times/week	48	(22.9)	335	(130)	
	2–3 times/week	38	(18.1)	346	(99)	
	4–6 times/week	22	(10.5)	364	(104)	
	≥1 time/day	56	(26.7)	350	(105)	

SD, standard deviation. The Mann–Whitney U test was used for comparison of means between two groups, and the Kruskal–Wallis test was used for three or more groups.

**Table 3:** Association between fruits and vegetable intake and skin carotenoid levels (*n* = 210).

		Partial Regression Coefficient	SE	(95% CI)	t	*p* Value	*p* for Trend
Crude model							
Number of vegetable dishes (dish/day)	Almost never	Reference					0.034
	1	5.86	26.9	(−47.1, 58.8)	0.22	0.828	
	2	12.8	29.2	(−44.8, 70.5)	0.44	0.662	
	3	58.6	36.5	(−13.5, 131)	1.60	0.110	
	≥4	83.1	53.8	(−22.9, 189)	1.55	0.124	
Frequency of green and yellow vegetables	≤1 times/week	Reference					0.796
	1 times/week	−21.5	43.3	(−107, 64.0)	−0.50	0.620	
	2–3 times/week	−9.86	39.8	(−88.3, 68.6)	−0.25	0.805	
	4–6 times/week	−23.5	40.7	(−104, 56.8)	−0.58	0.565	
	≥1 time/day	−13.0	42.6	(−97.0, 70.9)	−0.31	0.760	
Frequency of light-coloured vegetables	≤1 times/week	Reference					0.225
	1 times/week	62.0	34.6	(−6.16, 130)	1.79	0.074	
	2–3 times/week	58.0	32.4	(−5.99, 122)	1.79	0.075	
	4–6 times/week	98.3	34.4	(30.6, 166)	2.86	0.005	
	≥1 time/day	43.2	36.3	(−28.4, 115)	1.19	0.236	
Frequency of fruits intake	≤1 times/week	Reference					<0.001
	1 times/week	28.5	20.5	(−12.0, 69.0)	1.39	0.167	
	2–3 times/week	37.9	21.3	(−4.23, 79.9)	1.77	0.078	
	4–6 times/week	132	24.9	(83.1, 181)	5.31	<0.001	
	≥1 time/day	85.1	23.0	(39.8, 130)	3.70	<0.001	
Adjusted model ^†^							
Number of vegetable dishes (dish/day)	Almost never	Reference					0.035
	1	13.8	25.2	(−35.9, 63.5)	0.55	0.585	
	2	18.5	27.1	(−35.0, 72.0)	0.68	0.496	
	3	50.0	34.1	(−17.3, 117)	1.46	0.145	
	≥4	96.5	49.9	(−1.82, 195)	1.94	0.054	
Frequency of green and yellow vegetables	≤1 times/week	Reference					0.676
	1 times/week	17.2	40.5	(−62.6, 97.0)	0.42	0.672	
	2–3 times/week	32.3	37.7	(−42.2, 107)	0.85	0.394	
	4–6 times/week	20.2	38.4	(−55.5, 95.8)	0.53	0.600	
	≥1 time/day	30.7	40.3	(−48.9, 110)	0.76	0.448	
Frequency of light-coloured vegetables	≤1 times/week	Reference					
	1 times/week	52.8	32.2	(−10.6, 116)	1.64	0.102	0.236
	2–3 times/week	50.3	30.3	(−9.44, 110)	1.66	0.098	
	4–6 times/week	84.6	32.4	(20.8, 148)	2.61	0.010	
	≥1 time/day	39.8	34.1	(−27.4, 107)	1.17	0.244	
Frequency of fruits intake	≤1 times/week	Reference					<0.001
	1 times/week	24.8	19.5	(−13.8, 63.3)	1.27	0.207	
	2–3 times/week	30.7	20.3	(−9.45, 70.8)	1.51	0.133	
	4–6 times/week	112	23.7	(64.7, 158)	4.70	<0.001	
	≥1 time/day	64.3	22.2	(20.5, 108)	2.89	0.004	

SE, standard error; CI, confidence interval. Variables for fruit and vegetable intake were analysed in individual models. ^†^ Adjusted for sex, age, BMI, smoking status, alcohol drinking status, and dietary supplement use.

**Table 4:** Association between skin carotenoid levels and indicators of blood glucose levels and blood pressure.

	No. of Analysis Participants	Partial Regression Coefficient	SE	(95%CI)	t	*p* Value
Crude models						
log HbA1c	162	0.00012	0.00006	(0.000005, 0.00024)	2.05	0.042
log FBG	154	−0.00003	0.00010	(−0.00024, 0.00017)	−0.33	0.739
log SBP	183	−0.00005	0.00009	(−0.00024, 0.00013)	−0.58	0.565
log DBP	183	−0.00008	0.00010	(−0.00029, 0.00013)	−0.77	0.443
Adjusted model						
log HbA1c ^†^	162	0.00012	0.00005	(0.00002, 0.00022)	2.29	0.023
log FBG ^†^	154	0.00010	0.00010	(−0.00009, 0.00030)	1.04	0.299
log SBP ^‡^	183	−0.00014	0.00009	(−0.00032, 0.00004)	−1.54	0.126
log DBP ^‡^	183	−0.00014	0.00010	(−0.00034, 0.00005)	−1.43	0.154

SE, standard error; CI, confidence interval; HbA1c, glycated haemoglobin A1c; FBG, fasting blood glucose; SBP, systolic blood pressure; DBP, diastolic blood pressure; BMI, body mass index. Variables for indicators of glucose metabolism and blood pressure were analysed in individual models. ^†^ Adjusted for sex, age, BMI, smoking status, alcohol drinking status, dietary supplement use, and history of diabetes. ^‡^ Adjusted for sex, age, BMI, smoking status, alcohol drinking status, dietary supplement use, and history of hypertension.

## Data Availability

The data presented in this study are available on request from the corresponding author. The data are not publicly available due to privacy and ethical concerns.
